# An Invasive Disease Event-Free Survival Analysis to Investigate Ki67 Role with Respect to Breast Cancer Patients’ Age: A Retrospective Cohort Study

**DOI:** 10.3390/cancers14092215

**Published:** 2022-04-28

**Authors:** Raffaella Massafra, Samantha Bove, Daniele La Forgia, Maria Colomba Comes, Vittorio Didonna, Gianluca Gatta, Francesco Giotta, Agnese Latorre, Annalisa Nardone, Gennaro Palmiotti, Davide Quaresmini, Lucia Rinaldi, Pasquale Tamborra, Alfredo Zito, Alessandro Rizzo, Annarita Fanizzi, Vito Lorusso

**Affiliations:** 1 Struttura Semplice Dipartimentale di Fisica Sanitaria, I.R.C.C.S. Istituto Tumori “Giovanni Paolo II”, Viale Orazio Flacco 65, 70124 Bari, Italy; r.massafra@oncologico.bari.it (R.M.); s.bove@oncologico.bari.it (S.B.); v.didonna@oncologico.bari.it (V.D.); p.tamborra@oncologico.bari.it (P.T.); a.fanizzi@oncologico.bari.it (A.F.); 2 Struttura Semplice Dipartimentale di Radiologia Senologica, I.R.C.C.S. Istituto Tumori “Giovanni Paolo II”, Viale Orazio Flacco 65, 70124 Bari, Italy; d.laforgia@oncologico.bari.it; 3 Dipartimento di Medicina di Precisione, Università degli Studi della Campania “Luigi Vanvitelli”, 80131 Napoli, Italy; gianluca.gatta@unicampania.it; 4 Unità Operativa Complessa di Oncologia Medica, I.R.C.C.S. Istituto Tumori “Giovanni Paolo II”, Viale Orazio Flacco 65, 70124 Bari, Italy; f.giotta@oncologico.bari.it (F.G.); a.latorre@oncologico.bari.it (A.L.); vitolorusso@me.com (V.L.); 5 Unità Opertiva Complessa di Radioterapia, I.R.C.C.S. Istituto Tumori “Giovanni Paolo II”, Viale Orazio Flacco 65, 70124 Bari, Italy; a.nardone@oncologico.bari.it; 6 Struttura Semplice Dipartimentale di Oncologia Per la Presa in Carico Globale del Paziente, I.R.C.C.S. Istituto Tumori “Giovanni Paolo II”, Viale Orazio Flacco 65, 70124 Bari, Italy; g.palmiotti@oncologico.bari.it (G.P.); l.rinaldi@oncologico.bari.it (L.R.); rizzo.alessandro179@gmail.com (A.R.); 7 Unità Operativa Complessa di Anatomia Patologica, I.R.C.C.S. Istituto Tumori “Giovanni Paolo II”, Viale Orazio Flacco 65, 70124 Bari, Italy; a.zito@oncologico.bari.it

**Keywords:** ki67, invasive breast cancer, IDEFS, survival analysis, Kaplan–Meier

## Abstract

**Simple Summary:**

With the aim of enabling clinicians to design personalized therapeutic options according to patients’ age, in this study we investigated the relation between different threshold values of ki67, involved for defining breast cancer molecular subtypes along with other prognostic biomarkers, and the predisposition to develop a breast cancer-related invasive disease event (IDE) at different ages. As a result, our work shows how two different values of ki67, i.e., 10% and 20%, might be discriminant for the assignment of oncological therapies for patients under 50 years old and over 50 years old, respectively.

**Abstract:**

Characterization of breast cancer into intrinsic molecular profiles has allowed women to live longer, undergoing personalized treatments. With the aim of investigating the relation between different values of ki67 and the predisposition to develop a breast cancer-related IDE at different ages, we enrolled 900 patients with a first diagnosis of invasive breast cancer, and we partitioned the dataset into two sub-samples with respect to an age value equal to 50 years. For each sample, we performed a Kaplan–Meier analysis to compare the IDE-free survival curves obtained with reference to different ki67 values. The analysis on patients under 50 years old resulted in a *p*-value < 0.001, highlighting how the behaviors of patients characterized by a ki67 ranging from 10% to 20% and greater than 20% were statistically significantly similar. Conversely, patients over 50 years old characterized by a ki67 ranging from 10% to 20% showed an IDE-free survival probability significantly greater than patients with a ki67 greater than 20%, with a *p*-value of 0.01. Our work shows that the adoption of two different ki67 values, namely, 10% and 20%, might be discriminant in designing personalized treatments for patients under 50 years old and over 50 years old, respectively.

## 1. Introduction

Breast cancer has always been the most common malignant disease among women, and its molecular heterogeneity has made it difficult to design targeted treatments for each tumor type. Over the past 10–15 years, however, therapy concept has evolved, biological therapies have acquired more emphasis, and modern-day treatments have been driven by this molecular heterogeneity, which has allowed clinicians to classify breast cancer into subtypes [1]. Thus far, several studies have demonstrated the central role of breast cancer subtypes on both tumor prognosis and response to therapy [2,3,4]. However, despite the fact that proper breast cancer classification into subtypes should be realized by means of molecular testing, immunohistochemical (IHC) markers are commonly evaluated in clinical practice, due to both the elevated cost of and the limited access to molecular–genetic tests [5]. The most common IHC breast cancer prognostic markers are the hormonal receptors, comprising the estrogen receptor (ER) and the progesterone receptor (PgR), the human epidermal growth factor receptor-2 (HER2), and the cellular marker for proliferation ki67. The last-mentioned marker represents the percentage of labelled tumor cells within the investigated cell population, thus, the proliferative fraction of a cancer. Since it can be easily retrieved analyzing paraffin-embedded sections of tumor tissue, ki67 is a prognostic marker commonly used in clinical practice to determine tumor aggressiveness, and, consequently, to select the most appropriate breast cancer therapy [6].

According to these IHC markers’ expression, Italian guidelines provided a suitable classification of breast cancer subtypes and the related different treatment approaches [7].

The first-level classification involved IHC-based subtypes, such as Luminal-like, HER2-positive, and Triple-Negative tumors. Luminal-like tumors, which show a positive expression of the hormonal receptors, are the most common type of breast tumor, occurring in 60–70% of breast cancer patients. These tumors are also divided into two subgroups, A and B, based on the available percentage of cellular marker for proliferation. Particularly, Luminal B tumors, characterized by a ki67 expression higher than 20%, have a more aggressive nature and a less encouraging prognosis compared to Luminal A. On the other hand, HER2-positive and Triple Negative tumors are characterized by a negative expression of ER and PgR and suffer the worst prognosis [8].

Therefore, due to the hostile nature of Luminal B, HER2-positive, and Triple Negative breast cancers, patients affected by these tumors undergo chemotherapy [7]. Nonetheless, this treatment does not always allow a significant improvement in the overall and/or recurrence-free survival for pre-menopausal women [9]. By the way, different studies confirmed not only the positive association between a high expression of the cellular marker for proliferation and an early age, but also a worse prognosis for patients affected by breast cancer under these conditions [10,11].

Thus far, in the state-of-the-art, several studies have proposed time-to-event analysis for evaluating the occurrence of different events of interest in relation to specific prognostic factors [12,13,14].

In the work of Liang et al. [12], the authors performed a Kaplan–Meier analysis to estimate the recurrence-free survival of breast cancer patients belonged to different molecular subtypes and grouped into different subsamples with respect to both a low/high expression of ki67 and a different tumor grading.

The approach proposed in the work of Brandt et al. [13] aimed to evaluate breast cancer overall survival with respect to different age ranges, by means of a Cox Proportional-Hazard model. 

In our previous work [14], we performed a Kaplan–Meier analysis to evaluate and compare the disease-free survival of breast cancer patients treated with either breast conservative surgery or mastectomy.

Furthermore, many state-of-the-art studies have dealt with the relationship between breast cancer and ki67 [12,15,16,17], mainly focusing on either the lack of standardized procedures for ki67 assessment [15,16,17] or how the interplay of different prognostic factors affects recurrence-free survival in breast cancer patients [12].

However, to the best of our knowledge, there are not studies which examined the relation between different ki67 threshold values and the predisposition to develop a breast cancer-related invasive disease event (IDE), which includes local recurrence, the appearance of distant visceral and soft tissue metastases, contralateral invasive breast cancer, or a second primary tumor [18], at different ages. Besides, even though many studies have demonstrated that ki67 is a well-established prognostic marker, its role as predictive tool to identify patients who could benefit from a particular chemotherapy or endocrine treatment is not yet defined [16]. Specifically, there are not studies which assessed the predictive power of different ki67 values according to breast cancer patients’ age.

Therefore, in this study, we performed a Kaplan–Meier analysis to investigate the role of three different values of ki67 in estimating breast cancer-related invasive disease event-free survival (IDEFS) for patients under 50 years old and over 50 years old, with the aim of enabling clinicians to design personalized therapeutic options according to patients’ age.

## 2. Materials and Methods

### 2.1. Experimental Data

This retrospective study was approved by the Scientific Board of the Istituto Tumori “Giovanni Paolo II” of Bari, Italy, and involves only female patients with a first invasive breast cancer diagnosis in the period 1986–2020 who underwent adjuvant therapy and gave consent to use the data. The exclusion criteria were (a) patients who underwent neoadjuvant chemotherapy, (b) patients who had metastasis ab initio, and (c) patients who had carcinoma in situ. Finally, 900 patients, of which 603 non-IDE and 297 IDE, were enrolled. Particularly, among the IDE patients, local recurrences, contralateral cancers, distant metastasis, and second tumors were 72, 48, 147, and 30, respectively.

For each patient, clinical and histopathological data were collected, including age at diagnosis (abbr. age), previous tumors (values: absent/present), tumor size (abbr. diameter, values: T1a, T1b, T1c, T2, T3, T4), histological grade (abbr. grading, values: 1, 2, 3), histological type (values: ductal, lobular, other types), estrogen receptor expression (abbr. ER, % value), progesterone receptor expression (abbr. PgR, % value), cellular marker for proliferation (abbr. ki67, % value), human epidermal growth factor receptor-2 (abbr. HER2, values: positive/negative), tumor multifocality (abbr. multifocality, values: absent/present), angioinvasion (abbr. LVI, values: absent, focal, extensive, present but not typed), lymph node status (abbr. LN status, values: N0, N1, N2, N3). Moreover, for each patient, the related molecular subtype was assigned according to the St. Gallen consensus 2013 [19]. Specifically, patients characterized by a positive expression of the HER2 protein were classified as HER2-positive, whereas patients with a negative expression of the HER2 protein, as well as a negative expression of ER and PgR, were classified as Triple Negative. On the other hand, patients characterized by a negative expression of the HER2 protein and positive expression of the hormonal receptors were identified as Luminal-like and were assigned to either group A or group B with reference to a ki67 expression less or greater than 20%, respectively. Notably, a ki67 threshold value equal to 20% was retrospectively adopted for all patients. Finally, for each patient, chemotherapy information was acquired from medical records and the related chemotherapy regimen generations were assigned. Particularly, the first-generation regimen was assigned to patients who underwent a CMF scheme, the second-generation regimen was associated with patients who underwent an anthracycline-based chemotherapy, and the third-generation regimen was assigned to patients who underwent a chemotherapy scheme based on anthracycline and taxanes [20].

### 2.2. Statistical Analysis

With the aim of investigating the role of ki67 in the definition of treatment plans for breast cancer patients of different ages, the first step consisted of splitting the dataset into two sub-samples with respect to a specific age value, namely, 50 years, identified according to the state-of-the-art findings about breast cancer risk factors [21].

Subsequently, the two determined sub-samples were investigated by means of a Chi-square test, comparing the distributions obtained stratifying from time to time with reference to the clinical features collected.

Afterwards, in order to evaluate how different values of the cellular marker for proliferation affect the IDE-free survival in breast cancer patients aged under/over the previously selected cut-off, a Kaplan–Meier analysis was implemented on the two sub-samples, with respect to three ki67 threshold values, that is, 10%, 14% (according to the St. Gallen consensus 2009 [22]), and 20% (according to the St. Gallen consensus 2013 [19]).

The Kaplan–Meier estimator is the most common non-parametric approach for performing a time-to-event analysis, a method for evaluating the length of time until a well-defined, unequivocal, clinically significant, and easily diagnosable event of interest occurs [23,24]. The Kaplan–Meier method estimates the survival function S(t), which describes the probability of surviving over time, which is the probability that the event of interest has not yet occurred by a specific time point.

In case of incompletely observed survival times, we talk about censoring. The most common type of censoring, and the only one present in this study, is the so-called right censoring, namely, the phenomenon whereby some patients are lost to follow-up at one time point. Therefore, survival times are unknown for a sub-sample of individuals. Censored patients are involved for estimating survival probabilities at time points preceding their censoring time point, whereas they are excluded from the analysis thereafter [25].

A plot of the assessed survival function S(t) is provided by the Kaplan–Meier curve, a stepwise function in which every vertical drop represents the occurrence of one or more events, and the vertical marks denote right-censored patients at their censoring time [26].

When the survival functions of two or more different groups are confronted, a comparison of the related survival curves is possible by means of the log-rank test. Testing the null hypothesis that there is no difference in the probabilities of an event at any time point, the log-rank test returns a *p*-value as a dissimilarity measure among the Kaplan–Meier curves [27]. The compared distributions were considered statistically significantly different when the log-rank test returned a *p*-value less than 0.05.

Finally, with the aim of demonstrating IDEFS probabilities estimated by Kaplan–Meier were really affected by ki67, we performed a multivariate analysis with the Cox Proportional-Hazard model.

The Cox Proportional-Hazard model, which is a regression model commonly used for investigating the association between patients’ survival time and more predictive factors, estimates the cumulative hazard function H(t)=−log(S(t)) [28]. Before implementing the multivariate analysis, additionally, the few missing data reported in Table 1 were estimated by means of a missing data imputation algorithm, i.e., MissForest, a non-parametric method which can handle different types of variables concurrently [29]. Particularly, in order to reduce the parameters involved in the multivariate analysis, the following variables were sorted as follows: ki67 (values: 0–10, 11–20, 21–100), diameter (values: early, advanced), LN status (values: N0, N1, N2, N3), grading (values: G1, G2, G3), multifocality (values: absent, present), previous tumors (values: no, yes), histological type (values: ductal, lobular, others), LVI (values: absent, present).

All the analyses were performed by using R Statistical Software (v4.1.1., R Core Team 2021).

## 3. Results

Firstly, according to an age threshold value equal to 50 years, the starting dataset was partitioned into two sub-samples, on which all the subsequent analyses were performed.

The second step consisted of a statistical analysis of the sub-samples comprising 385 patients under 50 years old and 515 over 50 years old, respectively, by means of a Chi-square test. Particularly, for both sets, we estimated, and then compared in pairs, the normalized distributions obtained with respect to all the collected clinical features. Notably, only six classes of tumor size were considered, grouping together the sub-samples related to the T4 class, which occurs in a very low percentage in clinical practice. As summarized in Table 1, the distributions stratified as regards molecular subtype, LN status, multifocality, LVI, and chemotherapy had statistically significantly different results, with a *p*-value less than 0.05. On the contrary, the stratifications obtained with respect to diameter, grading, previous tumors, and histological type proved to be statistically associated. According to these results, the samples were considered as statistically different and, therefore, non-comparable throughout the further analysis.

Afterwards, a Kaplan–Meier approach was adopted with the aim of investigating the role of three different ki67 threshold values, namely, 10%, 14%, and 20%, in discriminating more and less aggressive breast cancers, with respect to patients’ age. Particularly, the analysis was performed on both sub-samples independently, and the IDEFS curves, along with two tables showing the number of patients at risk and the cumulative number of censorings over time, were computed for each examined cut-off. Besides, the median follow-up corresponds to 10 years for both sub-samples.

**Figure 1:**
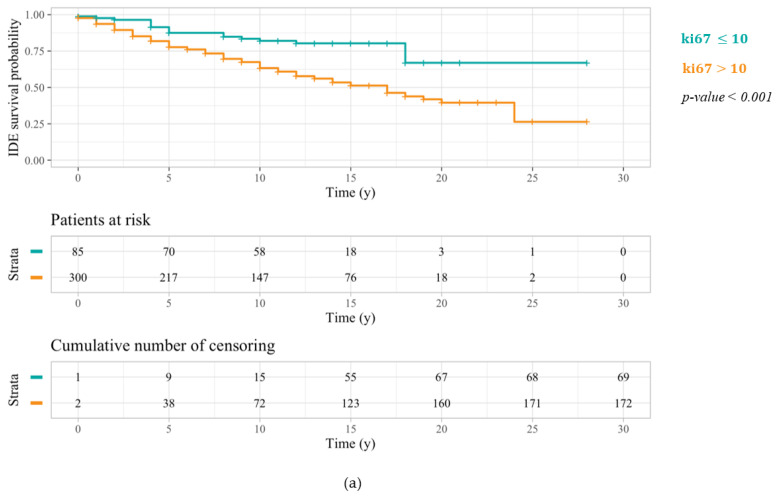

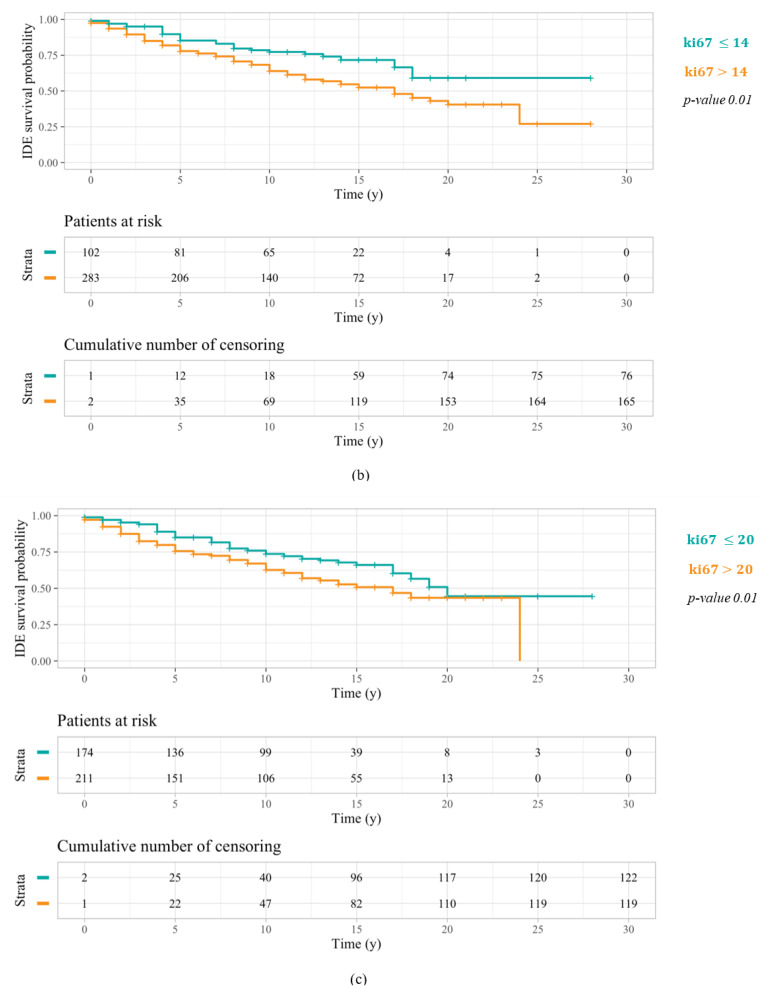
IDEFS curves related to a Kaplan–Meier analysis performed on patients under 50 years old with respect to the three different ki67 threshold values: 10% (**a**), 14% (**b**), and 20% (**c**). The log-rank test *p*-values highlighted a greater significance of the first cut-off.

Kaplan–Meier outcomes referred to patients under 50 years old and any one of the three ki67 threshold values are depicted in Figure 1. In every case, IDEFS curves of patients characterized by a ki67 greater than the selected threshold are located below the IDEFS curves of patients with a ki67 less than or equal to the cut-off under observation. Nonetheless, a ki67 threshold equal to 10% (Figure 1a) turned out to be the most statistically significant, returning a log-rank test *p*-value < 0.001, compared to the two other thresholds equal to 14% (Figure 1b) and 20% (Figure 1c), which returned a log-rank test *p*-value of 0.01.

**Figure 2:**
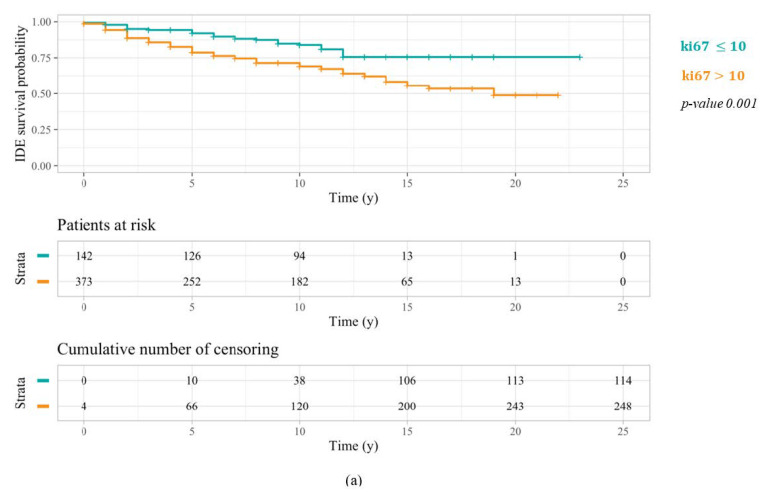

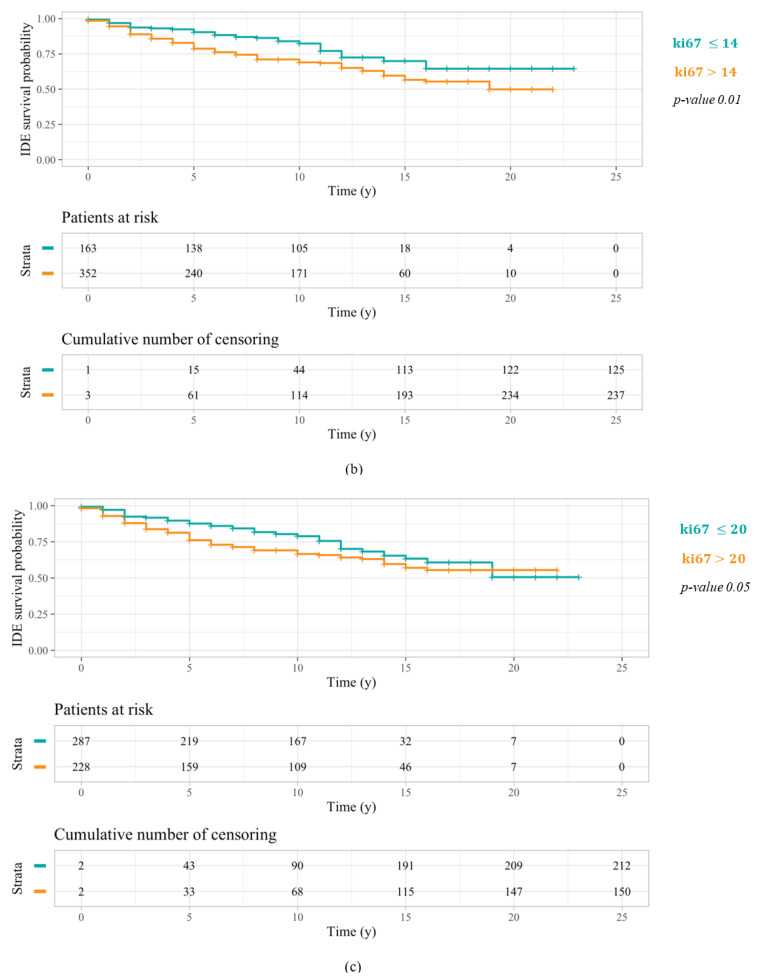
IDEFS curves related to a Kaplan–Meier analysis performed on patients over 50 years old with respect to the three different ki67 threshold values: 10% (**a**), 14% (**b**), and 20% (**c**). The log-rank test *p*-values highlighted a greater significance of the first cut-off.

As regards patients over 50 years old, the related Kaplan–Meier outcomes with respect to all three ki67 threshold values are illustrated in Figure 2. A clear distinction between the survival curve of patients characterized by a ki67 greater than the selected cut-off and even the one referring to patients with a ki67 less than or equal to the threshold under question is only evident in the first two cases. Actually, ki67 thresholds equal to 10% (Figure 2a) and 14% (Figure 2b) proved to be more statistically significant than a threshold equal to 20% (Figure 2c), thus returning log-rank test *p*-values of 0.001, 0.01, and 0.05, respectively.

**Figure 3:**
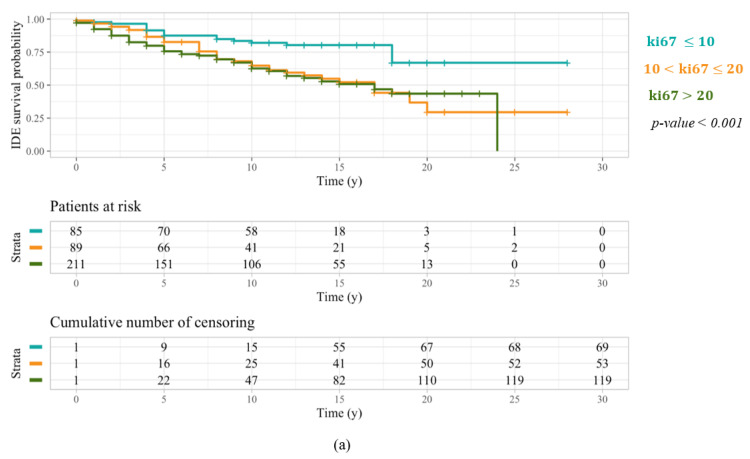

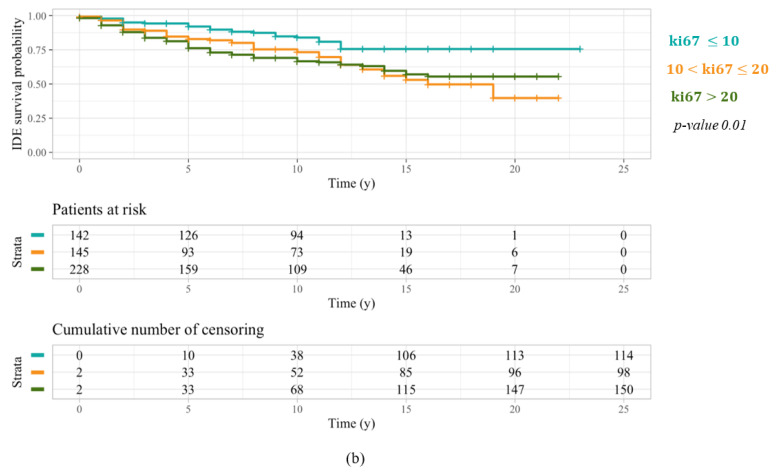
IDEFS curves related to a Kaplan–Meier analysis performed on patients under 50 years old (**a**) and over 50 years old (**b**), both stratified with respect to two ki67 threshold values: 10% and 20%. Similar behavior occurred for patients under 50 years old characterized by a ki67 ranging from 10% to 20% and greater than 20%. Conversely, patients over 50 years old with a ki67 ranging from 10% to 20% had an IDEFS probability greater than patients with a ki67 percentage greater than 20%.

Furthermore, as a more aggressive treatment is intended for HER2, Triple Negative, and Luminal B patients, we wanted to simultaneously compare the behavior of patients characterized by a ki67 ranging from 10% to 20%, and thus assigned to less invasive therapy plans in case of a positive expression of the hormonal receptors, with patients characterized by a ki67 expression less than 10% and greater than 20%. For this purpose, a further Kaplan–Meier analysis was performed on both sub-samples stratified into three groups with respect to these specific ki67 thresholds. As far as patients under 50 years old were concerned, similar behavior occurred for patients characterized by a ki67 ranging from 10% to 20% and greater than 20% (Figure 3a), showing that a percentage of cellular marker for proliferation greater than 10% is already related to a greater breast cancer aggressiveness, in younger patients. Meanwhile, patients over 50 years old characterized by a ki67 ranging from 10% to 20% showed an IDEFS probability greater than patients with a ki67 percentage greater than 20% (Figure 3b), revealing a reduced tumor aggressiveness in older patients with a major cellular marker for proliferation expression. These patients, frequently designated to a less invasive therapy, were characterized by a favorable prognosis for over 10 years. Besides, Table 2 summarizes 5-year and 10-year IDEFS probabilities for patients under 50 years old and over 50 years old stratified into three samples with reference to a ki67 equal to 10% and 20%.

**Figure 4:**
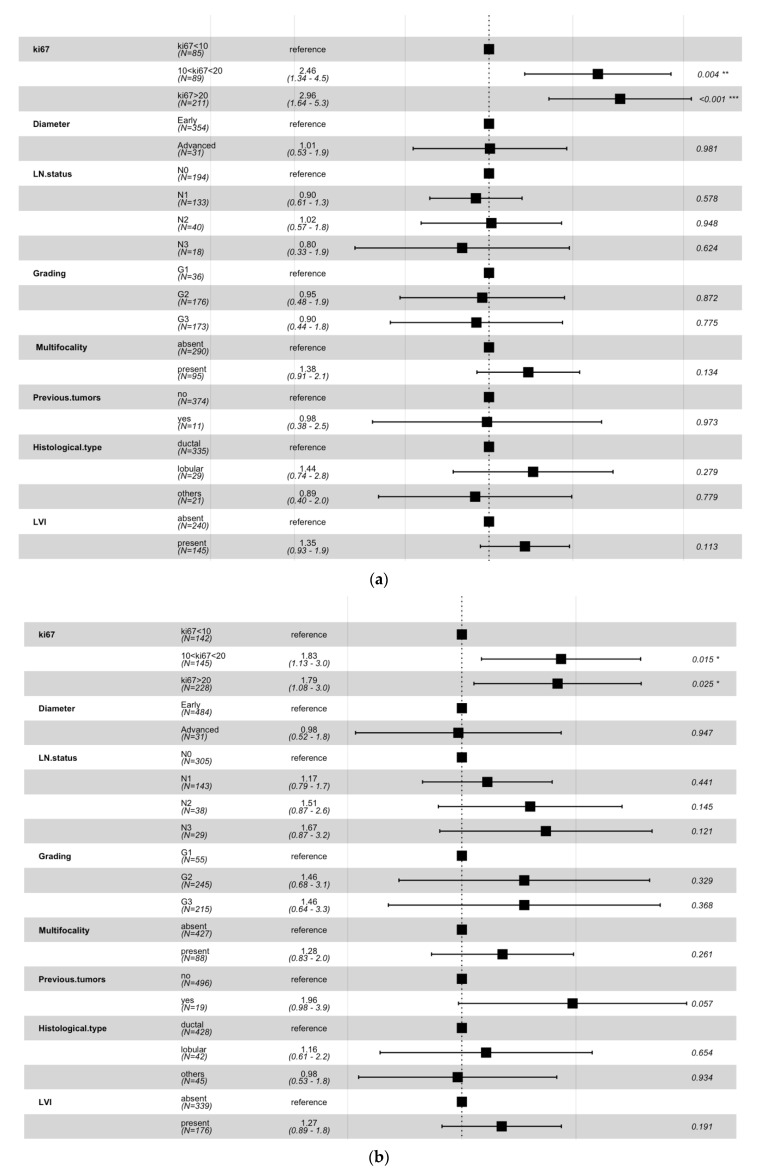
Multivariate analysis performed on patients under 50 years old (**a**) and over 50 years old (**b**) by means of the Cox proportional-hazard model. For each involved variable, the hazard rates (third column) and the *p*-values (last column) were computed by the model with respect to a specific reference value. * *p*-value < 0.05, ** *p*-value < 0.01, *** *p*-value < 0.001.

With the aim of demonstrating IDEFS probabilities estimated by Kaplan–Meier in the above-mentioned analysis were really affected by ki67, we performed a multivariate analysis with the Cox Proportional-Hazard model. For both patients under 50 years old (Figure 4a) and over 50 years old (Figure 4b), the multivariate analysis showed the only statistically significant factor was the cellular marker for proliferation expression.

**Figure 5:**
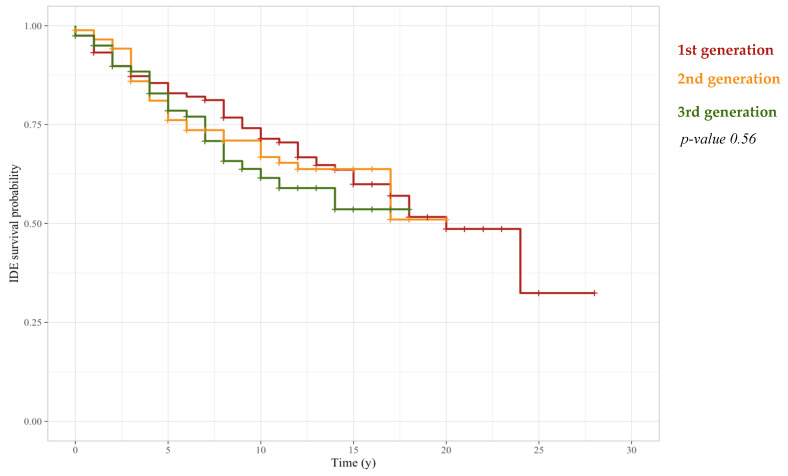
IDEFS curves related to a Kaplan–Meier analysis performed on patients under 50 years old stratified with respect to three different adjuvant chemotherapy generations: 1st generation CMF, 2nd generation Anthracycline, 3rd generation Taxanes/Taxanes and Anthracycline. The log-rank test demonstrated that there was not a statistically significant difference among the three populations.

As discussed above, a greater breast cancer aggressiveness occurs in younger patients characterized by a percentage of cellular marker for proliferation greater than 10%. Therefore, with the purpose of demonstrating that breast cancer care development over the years has not affected IDEFS probabilities estimated for patients under 50 years old, we performed a Kaplan–Meier analysis on these patients stratified with reference to chemotherapy schemes’ generations. Particularly, after estimating IDEFS probabilities, we observed that these probabilities were not affected by the adopted chemotherapy scheme, according to a *p*-value equal to 0.56 (Figure 5).

**Figure 6:**
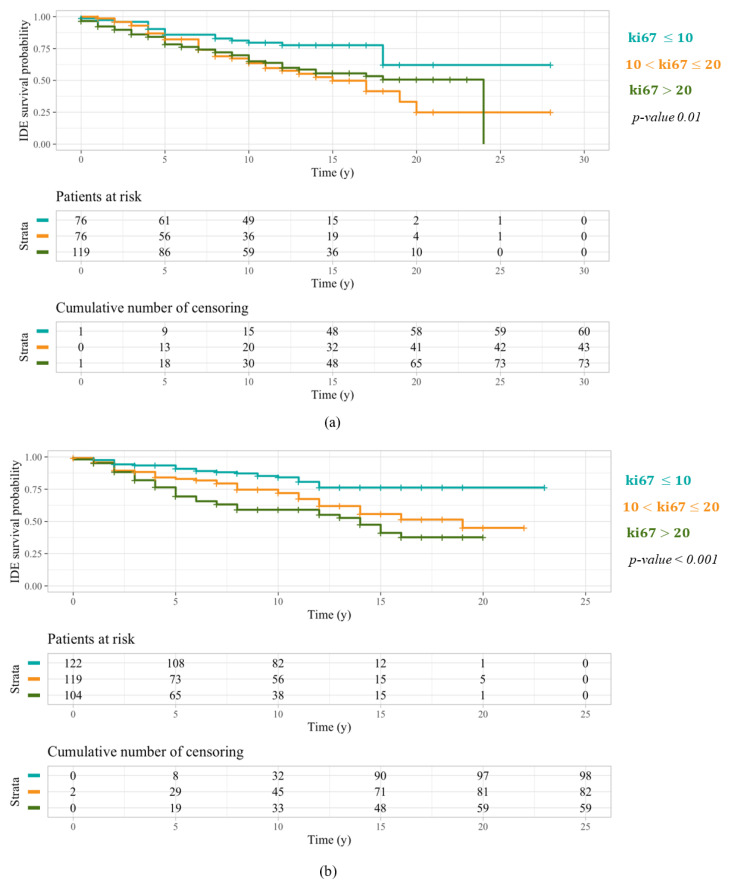
IDEFS curves related to a Kaplan–Meier analysis performed on Luminal-like patients under 50 years old (**a**) and over 50 years old (**b**), both stratified with respect to two ki67 threshold values: 10% and 20%. Luminal-like patients under 50 years old with a ki67 ranging from 10% to 20% showed a long-term IDEFS probability less than Luminal-like patients under 50 years old with a ki67 greater than 20%. On the contrary, Luminal-like patients over 50 years old with a ki67 ranging from 10% to 20% had a long-term prognosis greater than Luminal-like patients over 50 years old with a ki67 greater than 20%.

Finally, since the above discussed results may have been influenced by the heterogeneity of molecular profiles in the sample, we focused on patients with a Luminal-like tumor. Actually, while IDEFS probabilities of both HER2-positive and Triple Negative patients may be affected by other prognostic factors, the assignment of oncological therapies for Luminal-like patients mainly depends on the cellular marker for proliferation expression. Thus, after defining two sub-sets of Luminal-like patients including 271 under 50 years old and 345 over 50 years old, respectively, we stratified both samples into three groups with respect to the same two values of ki67, i.e., 10% and 20%, and we compared their IDEFS curves. According to our previous findings, Figure 6a shows how Luminal-like patients under 50 years old characterized by a ki67 ranging from 10% to 20%, likely designated to a less invasive therapy, had a long-term IDEFS probability significantly less than Luminal-like patients under 50 years old with a ki67 greater than 20%, then assigned to a more aggressive oncological treatment. On the contrary, even though Luminal-like patients over 50 years old with a ki67 ranging from 10% to 20% have likely undergone a less invasive treatment, their IDEFS probability, and then their long-term prognosis, were significantly greater than Luminal-like patients over 50 years old characterized by a ki67 greater than 20% (Figure 6b).

## 4. Discussion

Over recent years, an increased awareness of breast cancer’s heterogenous nature has led to treatment personalization and optimization. As a matter of fact, the analysis of genes and/or other markers present within cancer cells has allowed clinicians to classify patients characterized by different molecular profiles and to tailor treatment according to the specificities of a single breast cancer type in each patient [30]. In clinical practice, breast cancer classification into subtypes is performed estimating the expression of main IHC prognostic markers, such as ER, PgR, HER2, and ki67, in cancer cells. Particularly, according to these IHC markers’ expression, four molecular subtypes have been identified, each one associated with a different prognosis and a specified treatment approach [5].

Although the introduction of personalized treatments has led to an improvement in breast cancer survival, the combination of a high expression of the cellular marker for proliferation and an early age has proved to be an adverse factor for breast cancer-related IDEFS [10,11].

Thus, the aim of this work was to examine different ki67 threshold values for identifying the most-positively associated with a better IDEFS, both in younger and in older patients.

Firstly, we determined two sub-samples partitioning the starting dataset according to an age threshold value equal to 50 years. Subsequently, we performed a statistical analysis to compare the distributions obtained, stratifying with reference to the most informative histopathological features. Afterwards, we adopted a Kaplan–Meier approach for determining the IDEFS curves of patients belonging to the two sub-samples, with respect to three different threshold values of ki67, i.e., 10%, 14%, and 20%. Then, we performed a multivariate analysis with the Cox Proportional-Hazard model with the aim of demonstrating that IDEFS probabilities estimated by Kaplan–Meier were really affected by ki67. Finally, we focused on patients with a Luminal-like tumor, in order to overcome the heterogeneity sample bias.

The two determined sub-samples, namely, patients under 50 years old and over 50 years old, comprised 385 and 515 breast cancer patients, respectively. Anyway, since the Chi-square test revealed a significant difference among the samples, subsequent findings were analyzed independently. 

Kaplan–Meier analysis performed for all patients under 50 years old suggested that a percentage of cellular marker for proliferation equal to or greater than 10% was related to a very aggressive breast cancer and a lower IDEFS probability. Particularly, we observed that patients under 50 years old characterized by a ki67 ranging from 10% to 20% had an IDEFS probability close to the IDEFS probability of patients with a ki67 expression greater than 20%, who always undergo chemotherapy due to their worst prognosis. Specifically, when considering only Luminal-like patients under 50 years old, whose therapy plan is determined according to ki67 percentage, patients with a ki67 ranging from 10% to 20% showed the same short-term IDEFS probability as all patients under 50 years old. However, their long-term IDEFS probability was significantly less than Luminal-like patients under 50 years old with a ki67 greater than 20%. As a matter of fact, Luminal-like patients with a ki67 greater than 20% were typically intended for adjuvant chemotherapy in addition to a 5-year endocrine treatment. Thus, their probability of developing a breast cancer-related IDE has been reduced over the long term. On the other hand, Luminal-like patients with a ki67 less than 20% were commonly designated to a 5-year endocrine treatment alone. Although this kind of therapy can produce great survival benefits in breast cancer patients [31], the endocrine therapy alone could not be enough in women characterized by a more aggressive breast cancer. Accordingly, a better IDEFS may be observed in Luminal-like patients under 50 years old with a ki67 ranging from 10% to 20%, designating for them more aggressive targeted treatments, including adjuvant chemotherapy. More generally, tailored therapy plans may be defined for all patients under 50 years old according to the detected percentage of cellular marker for proliferation.

On the other side, a threshold value equal to 20% was confirmed as the most appropriate in discriminating more and less aggressive breast cancers for patients over 50 years old. Actually, patients over 50 years old with a ki67 expression ranging from 10% to 20% were characterized by a favorable prognosis, showing an IDEFS probability greater than patients with a ki67 percentage greater than 20%, for the first twelve years. Particularly, when considering Luminal-like patients over 50 years old, thus excluding HER2-positive and Triple Negative patients who typically suffer a worst prognosis, patients with a ki67 expression ranging from 10% to 20% were characterized by a prognosis always significantly better than Luminal-like patients over 50 years old characterized by a ki67 greater than 20%. Therefore, the 5-year endocrine therapy alone, designated for Luminal-like patients over 50 years old with a ki67 less than 20%, allowed these patients to reduce their probability of developing a breast cancer-related IDE, without undergoing more aggressive treatments such as adjuvant chemotherapy.

In conclusion, even though the present study does not examine the contribution of all predictive biomarkers useful for matching patient-targeted therapies and preventing toxicity of standard (systemic) therapies that could impact our outcomes [32,33,34], it showed that a ki67 threshold value equal to 10% could be considered for identifying aggressive tumors, and, thus, designing targeted treatments to increase IDEFS probability, in all patients under 50 years old, and especially in Luminal-like patients whose therapy plan is determined according to the detected ki67 percentage.

## 5. Conclusions

In the last few years, the identification of different breast cancer molecular profiles, according to the expression of particular IHC markers, has allowed clinicians to design personalized treatments for improving patients’ prognosis and increasing survival probability. Nevertheless, sometimes in clinical practice, therapeutic options determined depending on biomarker expression, such as the cellular marker for proliferation ki67, do not involve breast cancer patients’ age. Thus, with the purpose of designing personalized oncological treatments aimed at improving long-term prognosis, our work suggests the adoption of two different ki67 threshold values, i.e., 10% and 20%, for patients under 50 years old and over 50 years old, respectively. In conclusion, these different ki67 thresholds, in combination with both other biomarkers and prognostic factors, might be discriminant for the assignment of oncological therapies in either doubtful or borderline cases.

## Figures and Tables

**Table 1:** Under 50 years old and Over 50 years old sample distributions with respect to the most relevant histopathological features in differentiating breast cancer.

Features	Under 50 Years Old	Over 50 Years Old	Features	Under 50 Years Old	Over 50 Years Old
**Overall**	385; 100%	515; 100%	**Grading**		
Molecular Subtype *			G1 (abs.; %)	34; 8.8%	54; 10.5%
Luminal A (abs.; %)	151; 39.2%	240; 46.5%	G2 (abs.; %)	167; 43.4%	232; 45%
Luminal B (abs.; %)	120; 31.2%	105; 20.4%	G3 (abs.; %)	168; 43.6%	210; 40.8%
HER2^+^ (abs.; %)	52; 13.5%	93; 18.1%	NA (abs.; %)	16; 4.2%	19; 3.7%
Triple Negative (abs.; %)	62; 16.1%	77; 15%	Multifocality *		
**Diameter**			Absent (abs.; %)	290; 75.3%	427; 82.9%
T1a (abs.; %)	7; 1.8%	21; 4.1%	Present (abs.; %)	95; 24.7%	88; 17.1%
T1b (abs.; %)	41; 10.7%	60; 11.7%	**Previous Tumors**		
T1c (abs.; %)	157; 40.8%	216; 41.9%	Yes (abs.; %)	11; 2.9%	19; 3.7%
T2 (abs.; %)	133; 34.5%	175; 34%	No (abs.; %)	374; 97.1%	496; 96.3%
T3 (abs.; %)	19; 4.9%	11; 2.1%	**Histological type**		
T4 (abs.; %)	12; 3.1%	20; 3.9%	Ductal (abs.; %)	335; 87.0%	428; 83.1%
NA (abs.; %)	16; 4.2%	12; 2.3%	Lobular (abs.; %)	29; 7.5%	42; 8.2%
LN status *			Others (abs.; %)	21; 5.5%	45; 8.7%
N0 (abs.; %)	194; 50.4%	305; 59.2%	LVI *		
N1 (abs.; %)	130; 33.8%	139; 27%	Absent (abs.; %)	240; 62.4%	339; 65.8%
N2 (abs.; %)	40; 10.4%	38; 7.4%	Focal (abs.; %)	52; 13.5%	94; 18.3%
N3 (abs.; %)	18; 4.7%	29; 5.6%	Extensive (abs.; %)	29; 7.5%	20; 3.9%
NA (abs.; %)	3; 0.7%	4; 0.8%	Not typed (abs.; %)	64; 16.6%	62; 12.0%
Chemotherapy *					
None (abs.; %)	100; 26%	218; 42.3%			
1st generation (abs.; %)	118; 30.6%	129; 25%			
2nd generation (abs.; %)	87; 22.6%	92; 17.9%			
3rd generation (abs.; %)	80; 20.8%	76; 14.8%			

* p-value Chi-square test < 0.05.

**Table 2:** Values of 5-year and 10-year IDEFS of patients under 50 years old and over 50 years old stratified into three samples with reference to two different thresholds of the cellular marker for proliferation ki67. The *p*-values resulting from the statistical analysis highlight a statistically significant difference between 10-year IDEFS probabilities of patients under 50 years old and over 50 years old.

**Variation**	**5-Year IDEFS**					
	**ki67 < 10**		**10 ≤ ki67 < 20**		**ki67 ≥ 20**	
	**n. Events**	**Pr. (95% C.I.)**	**n. Events**	**Pr. (95% C.I.)**	**n. Events**	**Pr. (95% C.I.)**
Under 50 years old	10	87.5 (80.5–95.1)	14	82.6 (74.7–91.4)	49	75.6 (69.8–81.8)
Over 50 years old	11	92.1 (87.7–96.7)	22	82.9 (76.6–89.7)	51	76.2 (70.7–82.2)
*p*-value	0.1		0.5		0.3	
	**10-year IDEFS**					
	**ki67 < 10**		**10 ≤ ki67 < 20**		**ki67 ≥ 20**	
	**n. Events**	**Pr. (95% C.I.)**	**n. Events**	**Pr. (95% C.I.)**	**n. Events**	**Pr. (95% C.I.)**
Under 50 years old	14	82.0 (73.9–91.1)	26	64.7 (54.5–76.8)	71	62.6 (55.0–70.0)
Over 50 years old	21	84.0 (77.9–90.5)	32	73.3 (65.7–81.8)	68	66.6 (60.3–73.6)
*p*-value	0.3		0.02 *		0.1	

* p-value < 0.05.

## Data Availability

The data presented in this study are available on request from the corresponding author. The data are not publicly available because are propriety of Istituto Tumori ‘Giovanni Paolo II’—Bari, Italy.
